# Binocular contributions to motion detection and motion discrimination during locomotion

**DOI:** 10.1371/journal.pone.0315392

**Published:** 2024-12-20

**Authors:** Hongyi Guo, Robert S. Allison

**Affiliations:** 1 Centre for Vision Research, York University, Toronto, ON, Canada; 2 Department of Electrical Engineering and Computer Science, York University, Toronto, ON, Canada; Justus Liebig Universitat Giessen, GERMANY

## Abstract

During locomotion, the visual system can factor out the motion component caused by observer locomotion from the complex target flow vector to obtain the world-relative target motion. This process, which has been termed flow parsing, is known to be incomplete, but viewing with both eyes could potentially aid in this task. Binocular disparity and binocular summation could both improve performance when viewing with both eyes. To separate the binocular disparity and binocular summation and analyse how they affect flow parsing, we tested detection and discrimination thresholds under three viewing conditions: stereoscopic, synoptic (binocular but without disparity) and monocular. Experiment 1 tested motion detection during simulated forward self-motion and when stationary. Experiment 2 and 3 tested motion discrimination in forward and backward self-motion and stationary conditions. We found that binocular disparity significantly improved detection thresholds and discrimination biases, at the cost of lower precision. Binocular summation only significantly improved detection thresholds when stationary. It did not significantly affect detection thresholds during locomotion, discrimination biases, or discrimination precisions. Our results indicated that both binocular summation and binocular disparity contribute to motion detection and motion discrimination, but they affect performance differently while stationary and during locomotion.

## Introduction

During locomotion, the directions of objects in the surrounding environment change, creating a motion pattern in our visual field, which is known as optic flow [1]. When an object in the scene also moves, its flow pattern is complex, due to both the object motion and the locomotion of the observer. Nevertheless, the target motion can still be accurately perceived by a moving observer. For example, a sports player can often intercept a moving ball successfully. The flow parsing hypothesis [2–4] suggests that our visual system decomposes the composite flow to estimate the scene-relative object motion. Since its proposal, multiple studies have reported results consistent with the flow parsing hypothesis [2, 4–10].

Flow parsing gain is defined as the ratio of the subtracted flow vector to the locomotion component of the target motion vector [6] (in simpler words, the self-motion component that is subtracted divided by the actual self-motion component). The flow parsing gain serves as a measure of flow parsing effectiveness [6, 8, 9]. When gain = 1, the flow parsing is complete and all the self-motion component is factored out, When gain = 0, no flow parsing occurs. The gain has been reported to be generally below one [6, 8–10] (except for extreme cases such as a very short observation period [11]), suggesting that the flow parsing is incomplete, at least in the experimental conditions of these studies. The flow parsing gain has been found to be unaffected by object speed or the optic flow speed [6].

In the flow parsing process, optic flow plays a primary role in estimating scene-relative object movements [7]. However, integration with other sensory inputs may occur as well. MacNeilage et al. [12] showed that even vestibular inputs can be utilised for flow parsing. The role of binocular disparity in flow parsing has also been investigated sporadically. In one of the earliest flow parsing studies, Rushton and Warren [3] used lateral self-motion to show that observers were able to detect and discriminate target motion during simulated locomotion. In their stimulus [3], the target (a probe) was rendered either closer or farther than the fixation dot, and to correctly parse the moving direction of the target, observers had to use the correct distance order between the fixation dot and the target dot. This is because a lateral head movement produces motion parallax in opposite directions for a target closer to the observer than fixation compared to one further away than the fixation. Binocular disparity was chosen as the cue to the depth order in their experiment. Their observers could discriminate the target motion direction, which indicated that the visual system can capitalise on binocular disparity in this task. However, the stimulus was carefully chosen so that binocular disparity was the only available cue that could indicate the relative distances among their conditions. In the real world and in a more realistic VR scene, there could be multiple distance and depth cues, and some are monocular cues, such as object size and object height in the visual field. It is unknown whether observers still rely on binocular disparity in more realistic scenes, where multiple and potentially redundant cues are available.

Flow parsing involves removing the self-motion flow component from the target flow vector. When the locomotor speed increases, the optic flow increases and thus what needs to be cancelled increases. For accurate flow parsing, the discounted flow vector should scale with optic flow speed, which in turn scales with the locomotion speed. This is indeed what the visual system does, as shown by Niehorster and Li [6]. Binocular disparity has been found to enhance vection strength, perceived locomotion speed and perceived flow speed [13, 14]. If stereoscopic viewing changes the perceived locomotor speed, then this might impact flow parsing if, for example, this estimated locomotor speed is used to compute the component to be subtracted. Thus, stereoscopic information could have an indirrect effect, even if the optic flow pattern is unchanged.

In sum, there is evidence that flow parsing is a multisensory task [11, 12] in which binocular disparity has a role [3]. The locomotion component of the flow vector varies with distance, which can be estimated from the disparity [13, 14]. However, to the best of our knowledge, no study has compared monocular and binocular viewing conditions to closely examine how binocular viewing affects flow parsing. Realistic, complex scenes provide more depth cues than binocular disparity. Moreover, besides the provision of depth and distance cues, there can be other ways in which binocular viewing affects perception. Binocular summation is the term used to describe the improved performance when perceiving with two eyes than with one eye, and its proposed mechanisms include probability summation, neural summation, and improved accommodation [15]. Thus, a performance difference between monocular and binocular viewing conditions could be caused by binocular summation, binocular disparity, or both. Our experiments aimed to measure the effects of binocular viewing while carefully separating the possible effects caused by binocular summation and binocular disparity.

## Experiment 1

In this experiment, participants detected and indicated the moving target among four possible candidates. We measured detection thresholds with an adaptive staircase method. We hypothesise that both binocular summation and binocular disparity benefit the motion detection task by reducing the detection thresholds.

### Materials and methods

#### Participants

Twelve participants, aged 19–54 years (M = 30, SD = 10.08, eight males, four females), were recruited. The recruitment period started 1 October 2021, and ended 31 December 2021. Participants were students and vision scientists from York University, including the authors. All participants, except for the authors, were naive to the purposes of the experiment. All participants had normal or corrected to normal visual acuity, and had stereo acuity better than 100 arcseconds (tested with the Stereo Fly Test from Stereo Optical). Their interpupillary distances (IPD) were measured, and the system was adjusted accordingly. Participants provided informed consent. The study was approved by the Office of Research Ethics (ORE), and followed extra health and safety guidelines during the COVID-19 pandemic.

#### Apparatus

**Fig 1:**
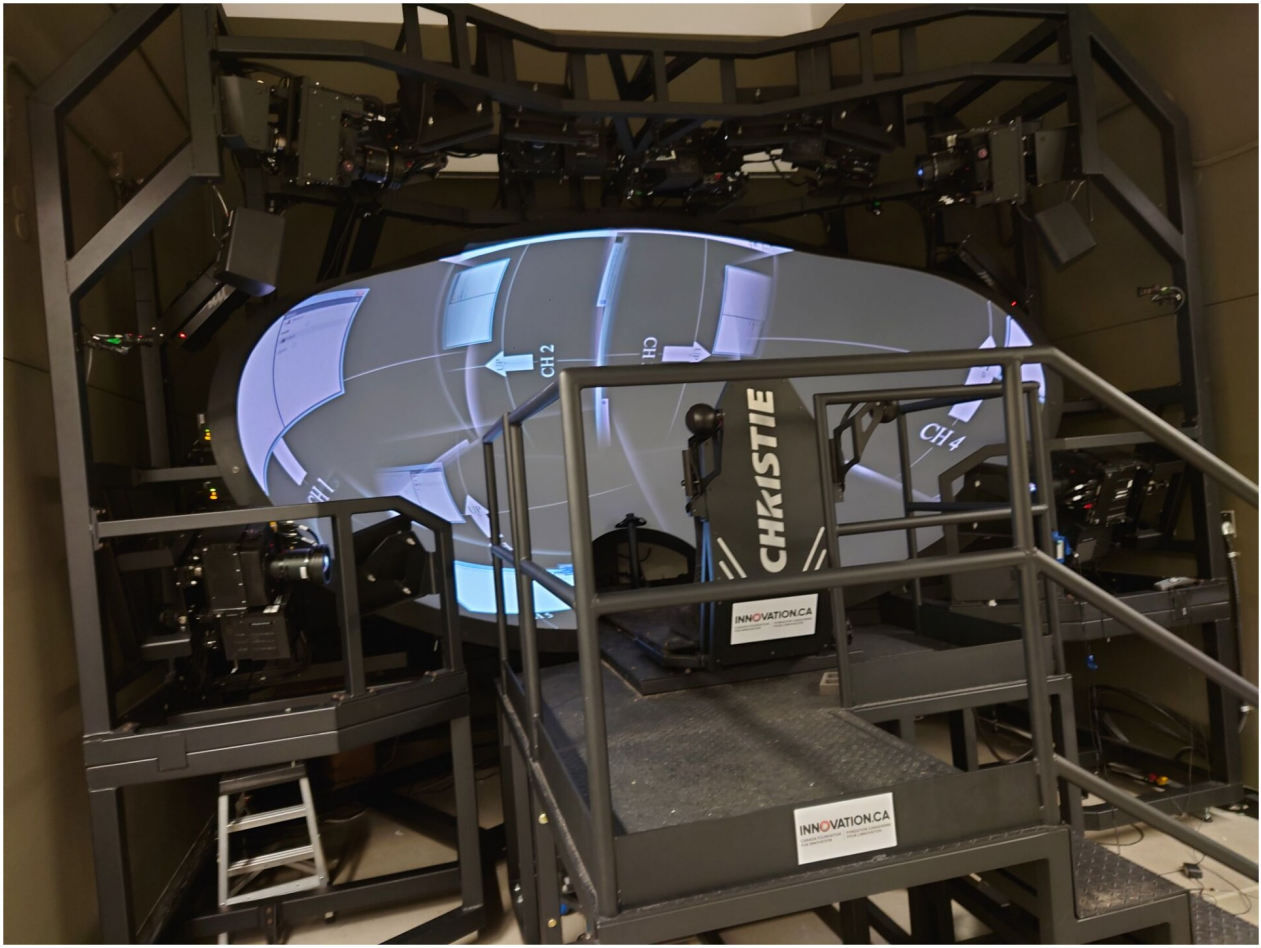
The wide-field stereoscopic environment (WISE) at York University. The user sits in the seat and views the projective screen, on which eight stereoscopic projectors project images at 120 Hz, or 60 Hz per eye when viewed stereoscopically with active shutter glasses. Almost invisible in the figure, six infrared sensors are also installed for head tracking or motion tracking. The seating platform can be removed and replaced with a standing platform or a treadmill, but for experiments in this paper the platform was in place as shown.

The experiment was conducted in the wide-field stereoscopic environment (WISE) at York University (Fig 1). WISE is a unique VR environment designed for vision experiments. It consists of a wide concave projective screen which covers almost the entire field of view of the user, and a movable platform with a viewer’s seat. The image on the projection screen was projected and hardware blended by 8 Christie Mirage WU-L stereoscopic projectors, each of them is controlled by a client computer (HP Z820 workstation with an nVidia Quadro k5000 graphics card). The projectors had a refresh rate of 120 Hz, which provides 60 Hz per eye when viewing stereoscopically with active shutter glasses. The lenses of active shutter glasses were opaque half of the time, so the luminance was greatly reduced. To optimise visual performance of the display and eliminate possible distractors, lights in the lab were turned off when WISE was in use. The frame timings among all projectors and the active shutter glasses were in hardware sync. Six WorldViz PPT infrared camera sensors tracked the two infrared markers mounted on the active shutter glasses. The observer’s head positions, computed by WorldViz PPT Studio, were passed to the VR software via VRPN (Virtual-Reality Peripheral Network) for real-time rendering based on the calculated eye-positions. The program for rendering the virtual environment was created with WorldViz Vizard, and the experiment was coded using Python 3 and the Psychopy package [16].

#### Stimuli and procedure

**Fig 2:**
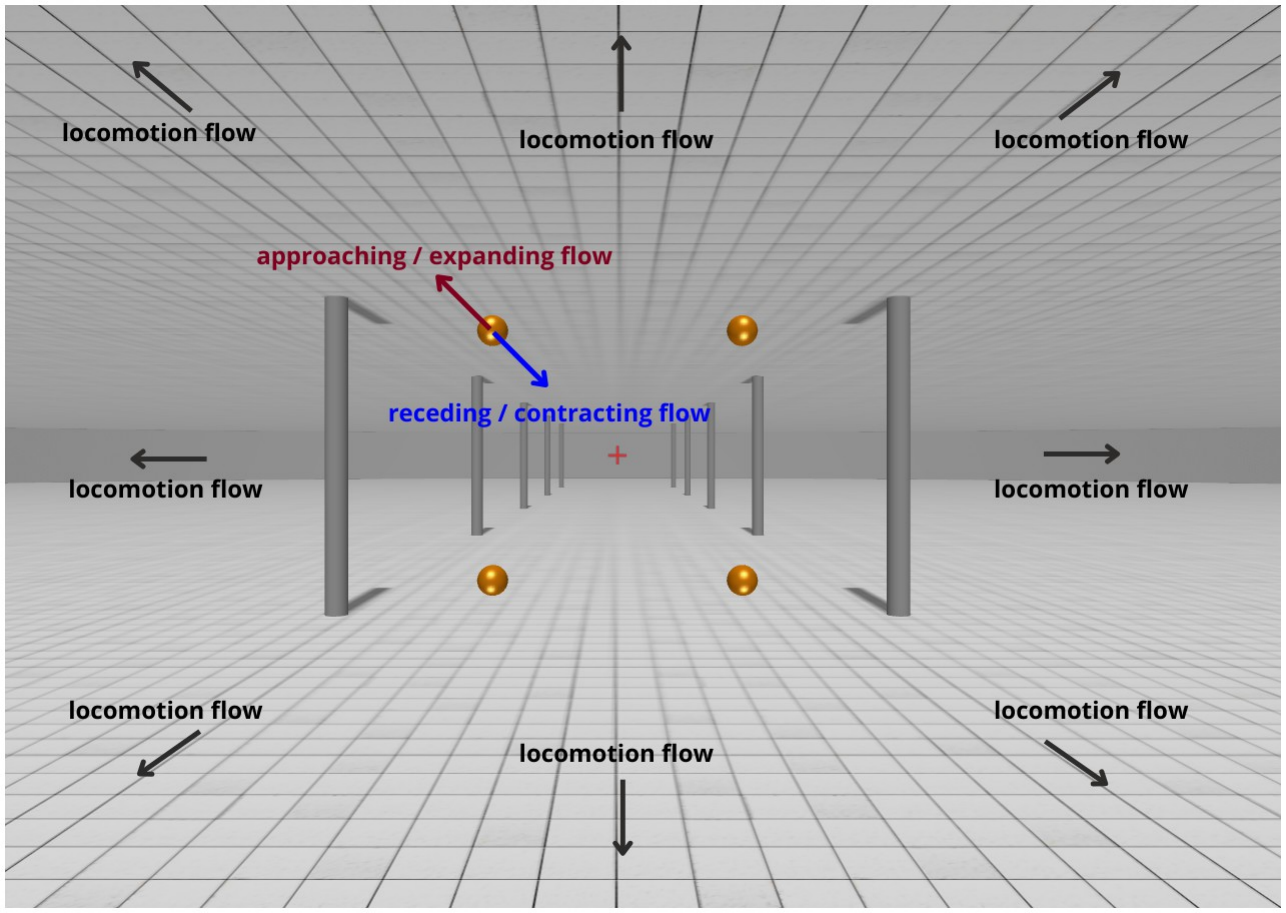
Stimulus of experiment 1.

An example stimulus is shown in Fig 2. Participants were presented with a virtual environment consisting of a background scene, four balls, and a fixation cross. On each trial, either a simulated forward locomotion or a stationary scene was presented for 0.5 s. The background scene was a hallway with a ceiling, a floor and pillars on both sides. The floor and ceiling had a grid pattern. The height of the ceiling was 3.4 m and the simulated eye height was 1.7 m. The fixation cross was placed at eye height in the centre of the observer’s FOV, 20 m in front of the observer. Participants were instructed to always keep fixation on the fixation cross whenever it was visible. Four golden balls (targets) which were 0.1 m in diameter, were placed in the four quadrants of the visual field, so that one target was inside each quadrant. Their distances to the sagittal plane and their distances to the horizontal plane that contains the cyclopean eye were both 0.4 m, and the initial distance to the frontoparallel plane that contained all four objects was 3 m. The eccentricity of each target was 10.7°, and the size was 3.7°, at the start of each trial. For a typical 60 mm IPD, the target has an initial horizontal binocular parallax of 0.95°, and the change in horizontal disparity due to locomotion would be 0.32° for a stationary target. On each trial, one of the targets moved relative to the scene in one of the following four directions:

- Approaching: moving in the direction which pointed from the front to the back of the observer.
- Receding: moving in the direction which pointed from the back to the front of the observer.
- Expanding: moving in the frontoparallel direction which pointed away from the centre of the participant’s visual field.
- Contracting: moving in the frontoparallel direction which pointed toward the centre of the participant’s visual field.

Since forward motion was simulated, a stationary, expanding or approaching target will move away from the focus of expansion (FOE) of the optic flow field. A receding or contracting target will only move toward the FOE if its speed is large enough, but this should rarely happen as the staircase converges to the threshold.

Each trial was divided into the following three phases. The next phase started immediately after the previous phase ended, and the next trial started immediately after the third phase of the previous trial ended.

1. Standby phase: During this phase, the background scene and the fixation cross were rendered. No target was visible, and the viewpoint was stationary. The duration of this phase was 0.5 second.
2. Motion phase: Once this phase started, all four targets appeared. A random target moved in one of the four possible directions. In the locomotion condition, the viewpoint started moving in the forward direction. The duration of this phase was also 0.5 second. The motion speed of the moving ball was controlled by the staircase algorithm (see below).
3. Response phase: The entire scene was covered with a grey colour, producing a blank display. The trial was paused until the participant responded. Then, this trial would end, and the next trial would begin immediately.

The task for the participants was to detect the moving target and report which one of the four targets moved during the trial. The probability of each target moving, and therefore chance performance, was 25%. The participants reported their choice by pressing the corresponding button on the controller. They did not need to specify which direction they saw the target moving. We used the threshold of the target motion speed for measuring participants’ motion detection performance. We expected this relationship to be monotonic within the range of motion used in this experiment: better performance corresponded to being able to detect the motion of the correct target under lower speed. We adopted an adaptive staircase algorithm, QUEST [17] to estimate the threshold. Since the task was a four-alternative forced choice (4AFC) task, the staircases targeted the 62.5% point on the psychometric function, which is the midpoint between 25%, guessing, and 100%, perfectly correct. One staircase of length of 50 trials was employed for each motion direction, making a total of 200 trials in a block. The staircases were interleaved randomly and the target to move was selected randomly, so that neither the moving target nor its moving direction was predictable. Participants were tested in two locomotion conditions:

- Forward locomotion: the viewpoint moved forward at 1.4 m/s during the motion phase. This will cause the scene-stationary targets to move away from the focus of expansion at approximately 6.28°/s.
- Stationary: the viewpoint was always stationary.

and three viewing conditions:

- Stereoscopic: the stereoscopic scene was rendered with a binocular disparity corresponding to the participant’s IPD.
- Synoptic: the same scene, which would be seen from a hypothetical central (cyclopean) nodal point, were presented to both eyes. Since the left eye and the right eye were presented the same images, no binocular disparity was introduced.
- Monocular: the right eye was presented the same scene as was presented in the stereoscopic condition; the left eye only saw a grey field, which had a similar brightness as the right scene.

Each participant was tested in all three viewing conditions and two locomotion conditions across six blocks. The order of the blocks was counterbalanced across participants to mitigate possible order effects. Before the first block, participants were given a practice of 10 trials to familiarize themselves with the task, under the same conditions as their first block. The participants were allowed to practice for more trials if they wished to. The experimental blocks were self-paced, and they were mostly finished within 10–15 minutes. Participants had a two-minute break in between the blocks. They were allowed to take a longer break if desired.

#### Data analysis

For each staircase, a threshold of target motion speed (*v*_1_, in m/s) was estimated as a measure of performance. To translate it into an angular metric, we obtained the angular motion of the target by calculating the angle (*α*_1_ in degree) between the egocentric direction of the target at the start and that at the end of the motion phase, if the target moved at *v*_1_. Similarly, let *α*_0_ be the angular motion of a scene-stationary target under the same locomotion. We define *α*_0_ and *α*_1_ to be positive when the movement vector points outward, and negative when it points inward. Directions can be assigned in this way because the optic flow patterns caused by target motions and locomotion were always radial. A simple example of a positive *α*_0_ and *α*_1_ is a scene-stationary target during a positive locomotion, in which *α*_0_ and *α*_1_ are both approximately 3.14°. The (angular) relative motion threshold (*rmt*) was defined as the following:

$$rmt=|\alpha_{1}-\alpha_{0}|$$

Thus, no matter whether the observer stays stationary or moves forward, *rmt* remains a measurement of target movement relative to the scene, and at the same time it is an angular term, suitable for studying visual perception.

We can also express the threshold in terms of angular speed by taking the *rmt* per second. Thus, the (relative) target motion speed threshold is:

$$\begin{array}{c} rmst=rmt/0.5=2rmt \end{array}$$ (1)

**Fig 3:**
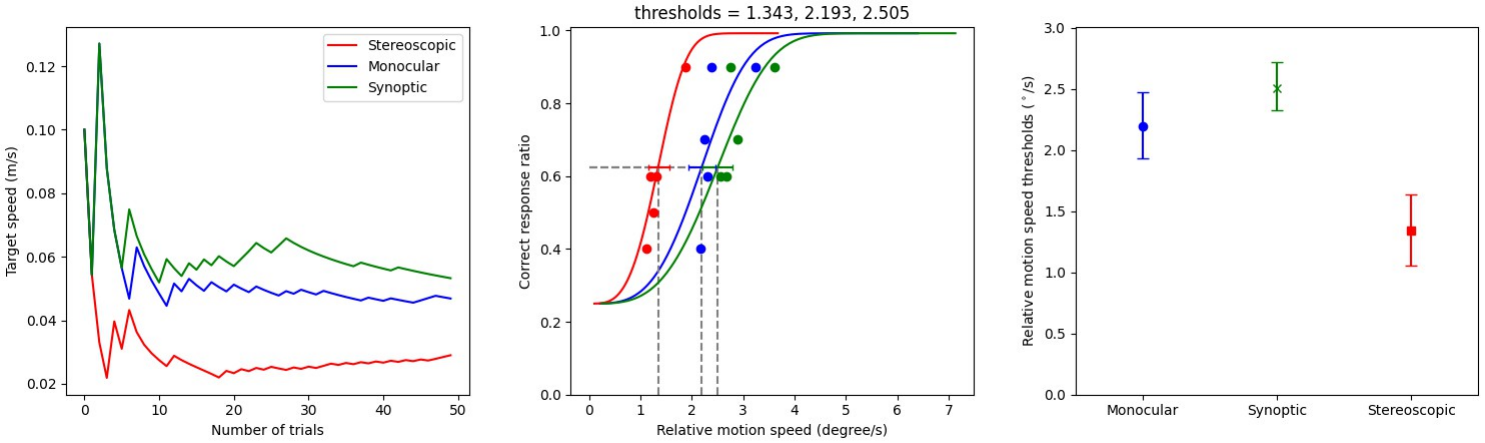
Sample result of experiment 1. This figure shows the data for contracting target movements, during forward locomotion of a participant. Left: The target motion speed as a function of trial number. Centre: The fitted psychometric functions in the three viewing conditions. Dashed lines show the fitted 62.5% thresholds. Each dot represents 10 trials. Right: Thresholds shown by viewing condition. Error bars show the 90% confidence intervals.

The thresholds that are mentioned in the following sections are all *rmst*, and their unit is °/s, unless otherwise specified. The threshold for each condition was calculated for each participant, and an example is shown in Fig 3. To analyse performance, the mean *rmst* across the locomotion and viewing conditions were compared. We expect that 1. the synoptic detection threshold is lower than the monocular detection threshold, and that 2. the stereoscopic detection threshold is lower than the synoptic detection threshold.

A three-factor (locomotion × direction × viewing condition) repeated-measures ANOVA and paired t-tests were performed for comparing means. The ANOVA and paired t-tests were performed in R with the anova_test and pairwise_t_test functions from rstatix package. We used the “auto” option provided by anova_test for correction of the degrees of freedom (DF). This means that the Greenhouse-Geisser correction was applied when the sphericity assumption was not met. The t-tests were two-sided, and the p values were adjusted with the Holm method.

### Results

#### Effects of target motion direction and its interaction with locomotion condition

**Fig 4:**
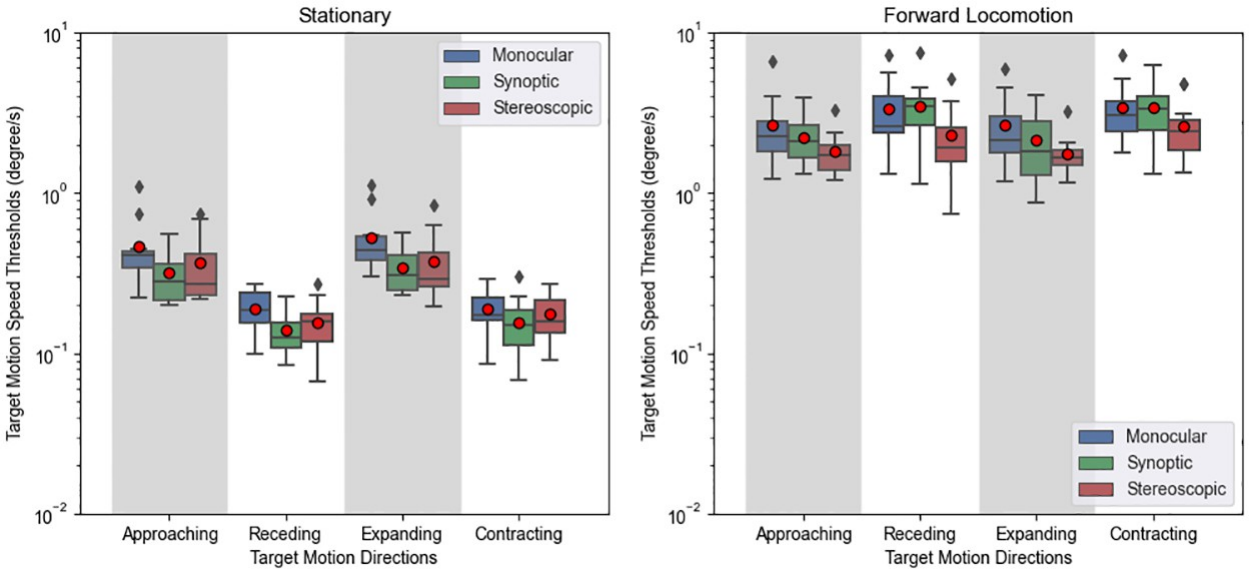
Result of experiment 1. Target detection thresholds, measured by taking the velocity difference between the moving target’s motion speed vector and the motion speed vector of a stationary target. Lower and upper box boundaries represent 25 (Q1) and 75th (Q3) percentiles respectively, line inside box shows the median and red dot represents the mean. Lower and upper error lines show Q1—1.5 interquantile range (IQR) and Q3 + 1.5 IQR. We use a logarithmic scale to show the data from both conditions.

As depicted in Fig 4, thresholds were much smaller when observers were stationary, indicating a strong main effect of locomotion, which was supported by the ANOVA test (F(1,11) = 102.028, p < .001, $\eta_{p}^{2}=0.903$). ANOVA results did not support a significant main effect of target motion direction (F(1.05,11.5) = 1.695, p = .219, $\eta_{p}^{2}=0.134$); however, there was a significant interaction between target motion direction and locomotion (F(1.06,11.66) = 5.995, p = .030, $\eta_{p}^{2}=0.353$).

**Fig 5:**
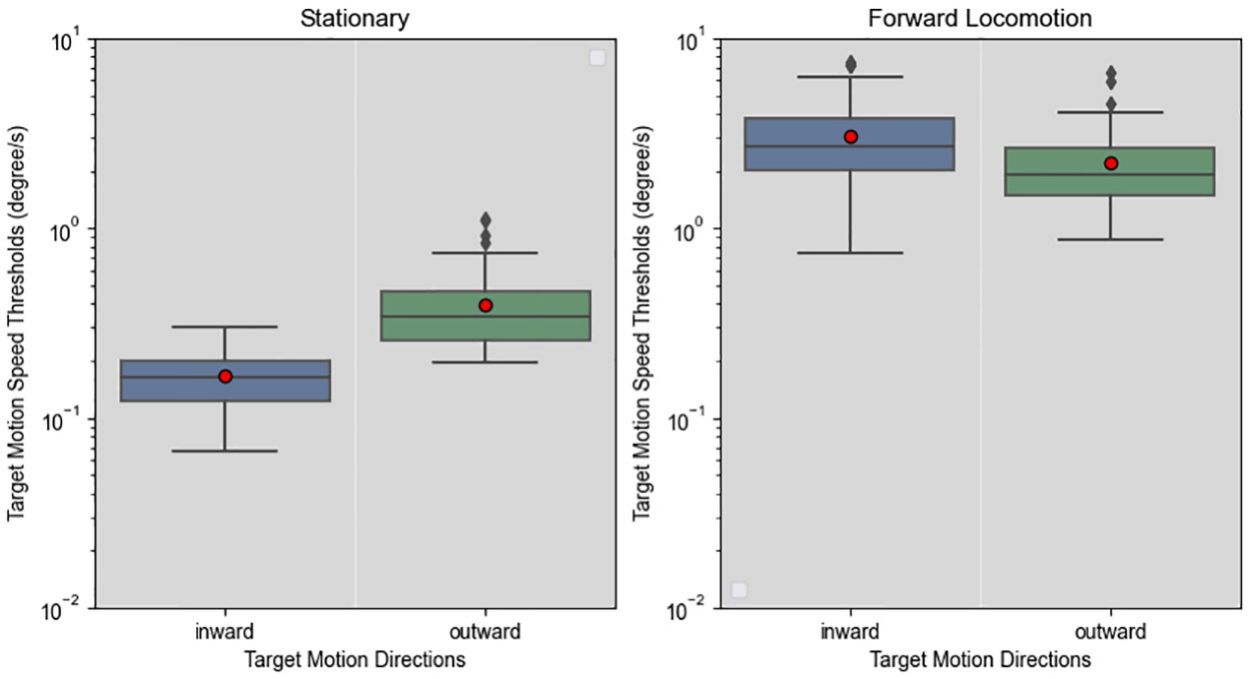
Grouping the motion directions in experiment 1. Target detection thresholds grouped by their optic flow direction. Approaching or expanding motions are classified as outward. Receding or contracting motions are classified as inward. When stationary, observers detected inward motions better, but during forward locomotion, they detected outward motions better. For explanation of box plot see Fig 4.

To analyse the interaction between directions and locomotion, the directions can be grouped either by the apparent optic flow patterns (contracting or expanding), or by the planes the direction vectors lie in (parasagittal or frontoparallel). Threshold comparisons by optic flow patterns are presented in Fig 5. Paired t-tests show that when stationary, inward motion thresholds were significantly lower (t(71) = 11.9, *p*_*adj*_ < .001) than outward motion thresholds. However, during forward locomotion, inward motion thresholds were significantly higher (t(71) = 4.24, *p*_*adj*_ < .001). While stationary, the more sensitive detection of centripetal motions we observed is consistent with previous studies [18, 19]. During forward locomotion, the observers were less sensitive to centripetal motions, possibly because the globally available expansive optic flow suppressed the responses of centripetal-tuned neurons. When comparing thresholds between parasagittal and frontoparallel target motions, the difference was not significant (stationary t(71) = .667, *p*_*adj*_ = .508; locomotion t(71) = .146, *p*_*adj*_ = .884).

#### Effects of viewing condition

**Fig 6:**
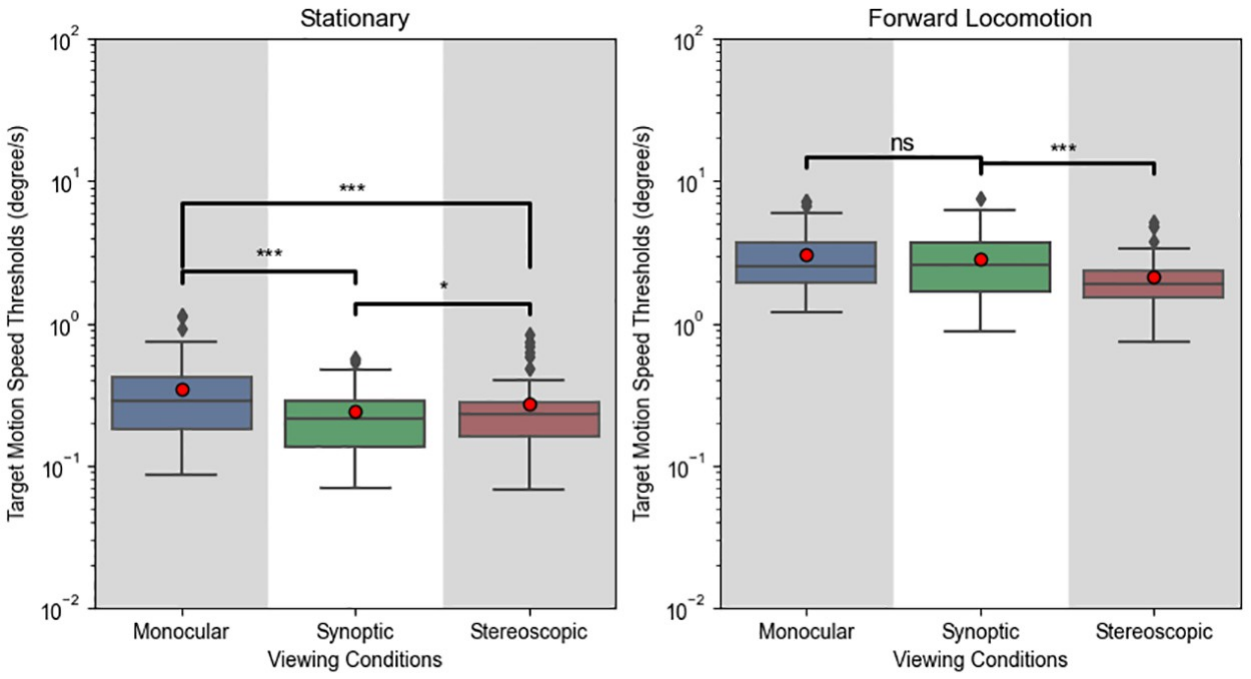
Experiment 1 results by viewing conditions. Refer to Fig 4 for explanation for box plot.

The results grouped by viewing condition are shown in Fig 6. ANOVA indicated a significant main effect of viewing condition (F(2,22) = 19.183, p < .001, $\eta_{p}^{2}=0.636$) as well as an interaction effect between viewing condition and locomotion (F(2,22) = 19.409, p < .001, $\eta_{p}^{2}=0.638$). No significant interaction between viewing condition and target motion direction was found (F(6,66) = 1.973, p = 0.082, $\eta_{p}^{2}=0.152$). Since we found an interaction, we performed paired t-tests separately for stationary and locomotion conditions. When stationary, the monocular threshold was higher than both synoptic (t(47) = 5.088, *p*_*adj*_ < .001) and stereoscopic (t(47) = 4.475, *p*_*adj*_ < .001) thresholds. The stereoscopic threshold was 12% higher than the synoptic threshold (t(47) = 2.565, *p*_*adj*_ < .014). However, during locomotion, monocular and synoptic did not differ significantly (t(47) = 1.829, *p*_*adj*_ = .074), but the stereoscopic threshold was 24% lower than the synoptic threshold (t(47) = -5.738, *p*_*adj*_ < .001), and 29% lower than the monocular threshold (t(47) = -6.342, *p*_*adj*_ < .001).

### Discussion

Tests of our main hypotheses and analysis of the interaction effect between the target motion direction and the locomotion condition suggests that (1) the participants were better at detecting inward (receding or contracting) motions when stationary, and (2) better at detecting outward (approaching or expanding) motions when moving forward. In our stimulus, when stationary, inward moving targets produced centripetal motion, while outward moving targets produced centrifugal motion. The first finding is consistent with the centripetal bias in sensitivity [18, 19]. In the forward-locomotion condition, an expanding optic flow was presented. The second finding is consistent with the study by Royden and Moore [20], which found that faster outward motions are detected more easily than slower outward motions in an expanding optic flow field.

Our finding of a facilitatory effect of binocular viewing was in line with existing evidence [3, 4] that binocular disparity can be integrated in the flow parsing task, and we extended it to linear forward locomotion. Furthermore, we separately examined the effect of binocular summation and binocular disparity. The observer’s performance could benefit from binocular viewing in at least two ways: binocular summation [15] of the monocular cues, and the integration of an extra depth cue—binocular disparity. We attempted to separate binocular disparity and binocular summation by testing three levels of viewing condition (monocular, synoptic and stereoscopic). The advantage of synoptic viewing over monocular viewing when stationary can be explained by binocular summation, and the advantage of stereoscopic viewing over synoptic viewing during locomotion can be explained by the facilitation from binocular disparity. Our results suggest that binocular viewing always had a significant facilitatory effect on motion detection regardless of locomotion conditions, but the cause of facilitation was different when stationary compared to during locomotion. The improvement, when stationary, reflects binocular summation. But during locomotion, it was binocular disparity. When stationary, all motion in the optic array was caused by object motion. The motion signals were available monocularly and could be strengthened by binocular summation. This can explain why binocular summation facilitated this task. The depth information inferred by binocular disparity would not be helpful when detecting relative image motion by comparison of the four motion signals. During locomotion, accurate flow parsing requires some information or assumption of the distance. For an observer moving forward at a linear speed *v*, the angular velocity *ω* of an object at an eccentricity of *ϕ* and a distance of *r* is:

$$\begin{array}{c} \omega =\frac{v\;\text{sin}\;\phi }{r} \end{array}$$ (2)

Thus, the optic flow vector *ω* is inversely proportional to the distance *r*. In the flow parsing process, *ω* is what needs to be factored out. Binocular disparity could improve the accuracy of *r* to enable a more accurate flow parsing.

## Experiment 2

In this experiment, the participant discriminated target motion directions during forward or backward locomotion, or when stationary. Similarly to experiment 1, we hypothesise that both binocular summation and binocular disparity facilitate the flow parsing task. To be specific, we hypothesise that there will be (1) an improvement in precision and accuracy between monocular and synoptic conditions, and (2) an improvement in precision and accuracy between synoptic and stereoscopic conditions.

### Materials and methods

The experiment 2 was conducted with the same apparatus as experiment 1.

#### Participants

Twelve participants aged 22 to 54 years (M = 29.8, SD = 9.47, 8 males, 4 females) were recruited the same way as in experiment 1, from 1 February 2022 to 31 March 2022. Eight participants also participated in experiment 1.

#### Stimuli and procedure

**Fig 7:**
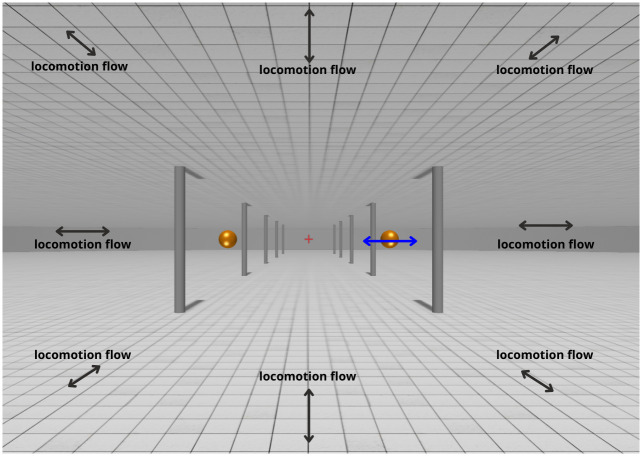
Stimulus of experiment 2.

An example scene is shown in Fig 7. The background scene of experiment 2, which was the same as that of experiment 1, included the pillars, the ceiling and the floor. In experiment 2, there was only one target (the golden ball) which appeared at eye-level, either to the right or to the left. On each trial, participants experienced a visual simulation of moving forward, backward, or staying stationary. For forward-moving trials, the target was initially 3 m away, with an eccentricity of 12.3°. In the backward-moving trials, the viewpoint started at the end position of the forward trials and moved backward. On the stationary trials, the viewpoint was kept at the midpoint of the start and end position of a forward-moving trial. This was to keep the average distance of the target the same across all three conditions. The trial procedure and the viewing conditions were the similar to experiment 1. However, the target in experiment 2 only moved parallel to the sagittal axis, either approaching or receding. The task was also different. In experiment 2 participants reported in which direction (approaching or receding) the target moved *relative to the scene* by pressing a corresponding button on the controller.

In experiment 2 we used transformed up-down staircases [21] to estimate thresholds. Two staircases were used for each condition. The “receding” staircase targeted the 71% receding response threshold with a two-up one-down staircase and started with a receding motion. The “approaching” staircase targeted the 29% receding response threshold and started with an approaching motion using a one-up two-down staircase. We chose this method because we were interested in both the bias and the precision (sensitivity) of the task. Bayesian adaptive staircase methods, such as QUEST [17] and Psi [22], converge quickly for finding bias thresholds [21], but they are not always suitable for estimating sensitivity [23]. With two thresholds obtained, we could estimate the bias by taking the mid-point and estimate the sensitivity by taking the difference.

Each staircase started at a random speed between 1 m/s and 2 m/s, and their step sizes started at 1 m/s and halved at each reversal. The staircases were terminated after both 40 trials and 7 reversals were completed.

#### Data analysis

A 3 × 3 (locomotion condition × viewing condition) repeated-measures ANOVA and paired t-tests were performed. While the target could move forward or backward, it could only move on one axis, and the point of subjective equality (PSE) should be unique for a fixed locomotion condition and viewing condition. Therefore, the target direction is not an independent factor in experiment 2, unlike experiment 1. The packages, correction and adjustment methods used were the same as experiment 1, unless specifically noted otherwise.

We converted the thresholds into angular terms as in experiment 1 (see Eq 1). Let *rmst*_29_ and *rmst*_71_ be the 29% and 71% thresholds, we can define our bias and precision as the following:

$$bias\left(signed\right)=sign*\left(rmst_{29}+rmst_{71}\right)/2$$

$$precision=|rmst_{29}-rmst_{71}|/2$$

**Fig 8:**
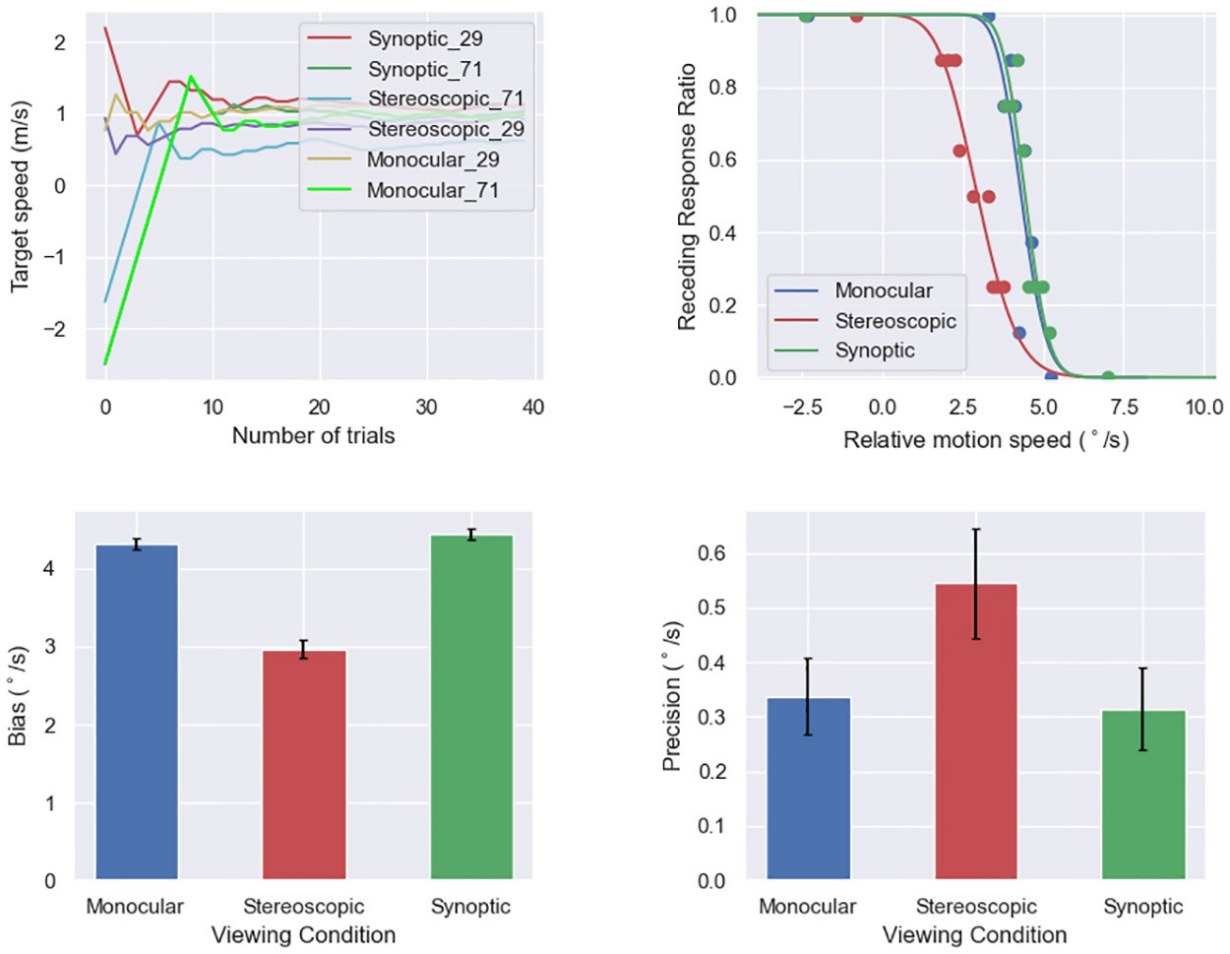
Sample result of experiment 2. This figure shows example data of a participant during backward locomotion. Top left: The target motion speed as a function of trial number. The ordinate axis is the scene-relative speed, which can be either positive or negative. Positive means the target moves in the direction towards the observer in the scene. Top right: The fitted psychometric functions in the three viewing conditions. Each dot represents data from 10 trials. Note that the abscissa shows egocentric motion speed, and positive means outward. Bottom left: Biases. Bottom right: Precision (sensitivity). Error bars show 90% confidence intervals.

Since the *rmst* were defined as speed thresholds without directions, and we have two directions (approaching and receding) in experiment 2, we added a sign for bias. We define *sign* = 1 if the bias is outward (relative to the flow vector of a stationary target), and *sign* = −1 if the bias is inward. We observed that in our results, forward locomotion always induced an inward (thus negative) bias and backward locomotion always induced an outward (positive) bias. An example result is shown in Fig 8. When comparing bias magnitudes, the sign of the bias was flipped for the forward locomotion condition to enable comparison of magnitudes amongst the condition. In the following subsections, both bias and precision comparisons are in angular terms.

### Results

#### Effects of locomotion direction

**Fig 9:**
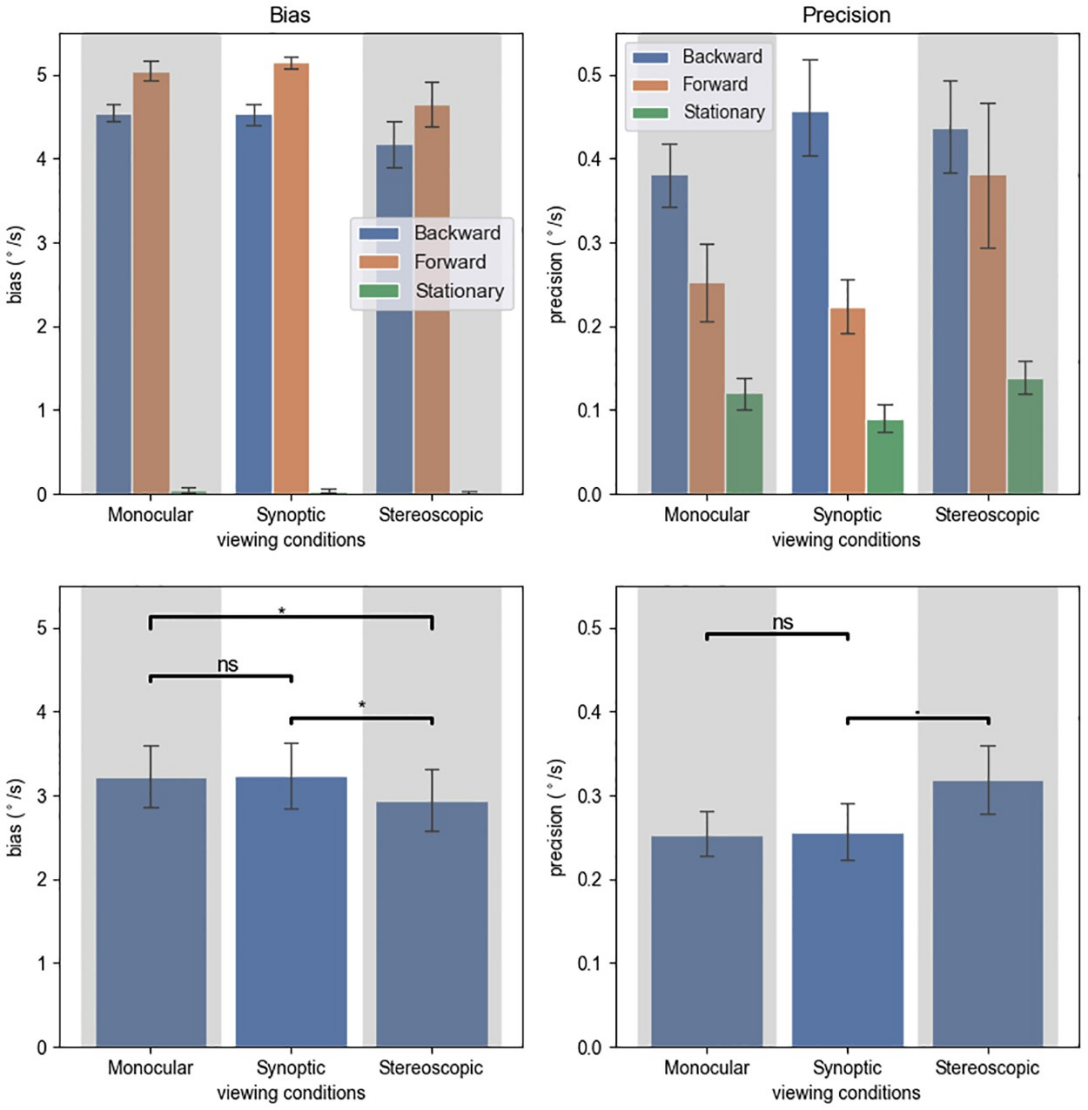
Experiment 2 results by viewing conditions. Top left: biases, which were calculated by taking the average of the 29% and 71% thresholds. Top right: precision, obtained by taking half of the difference between the 29% and 71% thresholds. Bottom: biases and precisions by viewing conditions, with significance annotations. “.”: .05 ≤ p < .1, “*”: .01 ≤ p < .05. Error bars represent standard errors.

The results are depicted in Fig 9. The ANOVA test showed a significant main effect of locomotion condition on bias (F(2,22) = 714.693, p < .001, $\eta_{p}^{2}=0.985$). The F value is extremely large, because in the stationary condition, the biases were very close to zero, showing an exceptionally high accuracy for motion discrimination compared to the other two conditions (Forward—Stationary: t(35) = 43.954, *p*_*adj*_ < .001; Backward—Stationary: t(35) = 41.002, *p*_*adj*_ < .001). Moreover, forward biases were slightly (11.97%) greater than backward biases (t(35) = 6.946, *p*_*adj*_ < .001).

A significant main effect of locomotion condition (F(1.31,14.64) = 23.361, p < .001, $\eta_{p}^{2}=0.680$) on precision was revealed by the ANOVA. The stationary precision was better (59.44% smaller in value) than the forward precision (t(35) = 4.930, *p*_*adj*_ < .001), and the forward precision was better than (32.86% smaller in value) than the backward precision (t(35) = 3.746, *p*_*adj*_ < .001).

#### Effects of viewing condition

Our hypotheses require a planned contrast among the conditions. We found that the stereoscopic bias was 8.40% lower than the monocular bias (t(35) = 2.803, *p*_*adj*_ = .025) and 9.22% lower than the synoptic bias (t(35) = 2.731, *p*_*adj*_ = .025), but we did not find a significant difference between the synoptic and monocular conditions (t (35) = .841, *p*_*adj*_ = .406). However, ANOVA did not support a significant effect of the variable (viewing condition) on bias, if we choose *α* = .05 (F(1.07,11.79) = 4.116, p = .063, $\eta_{p}^{2}=0.272$).

The interaction effect on bias between locomotion and viewing conditions was not significant (F(2.01, 22.16) = 2.359, p = .117, $\eta_{p}^{2}=0.177$).

Viewing condition had a significant main effect on precision (F(2, 22) = 3.672, p = .042, $\eta_{p}^{2}=0.250$). Specifically, stereoscopic precision was significantly worse (26.6% greater in value) than monocular precision (t(35) = 2.674, *p*_*adj*_ = .011). The stereoscopic precision was also worse than synoptic precision, but this difference was not significant (t(35) = 1.829, *p*_*adj*_ = .076). Synoptic and monocular precisions were not significantly different (t(35) = .192, *p*_*adj*_ = .849).

We did not find a significant interaction effect on precision between viewing condition and locomotion condition (F(2.46,27.08) = 2.287, p = .111, $\eta_{p}^{2}=0.172$).

### Discussion

#### The bias and the flow parsing gain

The large bias indicates that the background motion caused by locomotion was not fully compensated when parsing the target motion. Our definition of bias is linked to the flow parsing gain [6, 8, 9]. The flow parsing gain *g* was defined by Niehorster and Li [6] as the following (note that symbols are changed):

$$\begin{array}{c} g=1-\frac{|\omega_{a}|}{|\omega_{f}|} \end{array}$$ (3)

where *ω*_*f*_ is the self-motion component of the target’s motion. In other words, it is the target optic flow if the target were actually stationary relative to the scene. *ω*_*a*_ is the “nulling component” which has to be added into the stimulus to nullify the apparent target motion in the direction of *ω*_*f*_. Both *ω*_*a*_ and *ω*_*f*_ are angular motion vectors, describe the rate of change in egocentric directions, and in our study they also describe retinal motions because the eyes were fixated. A perfect observer fully compensates for *ω*_*f*_, thus has no bias, their |*ω*_*a*_| = 0, and *g* = 1.

Our bias is the same as *ω*_*a*_. Thus,

$$\begin{array}{c} g=1-\frac{\left|bias\right|}{|\omega_{f}|} \end{array}$$ (4)

**Fig 10:**
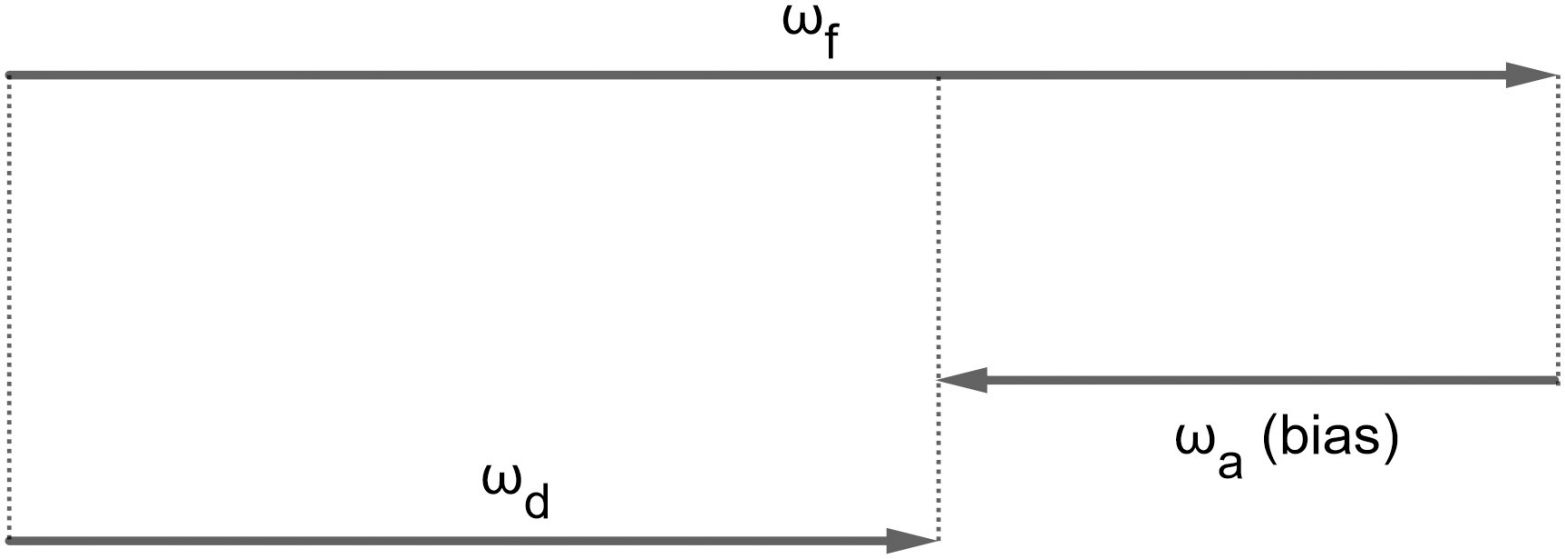
Motion vectors in flow parsing. The discounted flow vector, *ω*_*d*_, is the flow vector of a target when this target is perceived as scene-stationary. *ω*_*f*_ is the hypothetical flow vector if the target were stationary in the scene, or the “self-motion component” of the target motion. *ω*_*a*_ is the bias caused by scene-relative target motions, which can be obtained by subtracting *ω*_*f*_ from *ω*_*d*_. All vectors describe the motions or motion components of a single target. They were drawn in layers for visibility.

Let *ω*_*d*_ be the flow vector for a target at PSE. Thus, *ω*_*d*_ = *ω*_*f*_ + *ω*_*a*_ (as illustrated in Fig 10), and we have:

$$\begin{array}{c} g=\frac{|\omega_{d}|}{|\omega_{f}|} \end{array}$$ (5)

As one would imagine, *g* is defined for forward and backward locomotion conditions only, but undefined for the stationary condition, because |*ω*_*f*_| = 0. In experiment 2, *g*_*forward*_ = 21.07% and *g*_*backward*_ = 29.51%. Both are less than 1, consistent with the observation by Niehorster and Li [6].

The gains were much smaller than 1 (gain = 1 means perfectly accurate motion discrimination) because the biases were large. A large bias could be caused by an underestimated locomotion speed. For a slower locomotion (thus smaller *ω*_*f*_), assuming a roughly constant *g*, the visual system requires a smaller *ω*_*d*_ for it to perceive a stationary target. Thus, if the locomotion speed is underestimated, the target motion *ω*_*d*_ should be smaller than ideal. In our study, *ω*_*d*_ was determined by observer behaviour, but *ω*_*f*_ was calculated from the stimulus. Thus, an underestimated locomotion could cause a low gain *g* and a large bias *ω*_*a*_.

The bias could also be caused by an overestimated target distance. As explained earlier (see Eq 2), the magnitude of the optic flow vector is inversely proportional to the distance. That is to say, a farther target will have a smaller *ω*_*f*_. Assuming a roughly constant flow parsing gain, the visual system will perceive a smaller *ω*_*d*_ as stationary. Again, *ω*_*f*_ is the ideal value calculated from stimulus, so it would not scale with perceived distance. Thus, an overestimated target distance could also cause a low gain *g* and a large bias *ω*_*a*_.

It is worth noting that the underestimation of locomotor speed and overestimation of distance need not be consciously perceived by the observer. They can be “registered” properties (e.g. see the “registered distance” mentioned in [24, 25]), unconscious interpretations of the stimulus made by the visual system, or some assumptions taken by the visual system, for the purpose of performing flow parsing.

The PSE is the point where the target is equally likely to be perceived as moving forward or backward. Participants were instructed to only respond with either forward or backward. The target moved either forward and backward, with no lateral motion component, and indeed looked like it moved sagittally. However, forward and backward movements share the same flow pattern as lateral movement when the target is at eye level. Thus, the bias might also be induced by a component of the flow vector which was perceived as a lateral target motion.

#### The role of locomotion condition

The observers were much less accurate at direction discrimination with a non-zero optic flow field (consistent with either a forward or a backward locomotion), compared to when stationary. When stationary, there was hardly any bias. During locomotion, there was a bias in the locomotion direction (e.g., when moving forward, the target PSE was also in the forward, that is, the receding direction). Moreover, the backward bias was slightly smaller than the forward bias. The bias is the magnitude of vector difference between the perceived self-motion component and the true self-motion component of the target retinal motion. We intentionally kept the forward and the backward locomotion the same (with only swapping the start and end points), so that the target would have kept the same average distance if the biases were zero. It is worth noting that the biases in the locomotion direction caused the target to be closer on average during backward locomotion.

#### The role of binocular disparity

Binocular disparity significantly reduced the bias, but binocular summation did not. At the distance (about 3 metres) of the target, binocular disparity is a strong relative depth cue. The lack of binocular disparity in the non-stereoscopic conditions could diminish the perceived depth between the target and background and cause the target to look further away. As explained previously, an overestimated target distance associates to a greater bias.

Moreover, as we explained previously, the bias is consistent with an underestimated locomotion. It has been found that stereoscopic viewing can enhance vection strength and perceived self-motion speed [13, 14]. Our result of a reduced bias in the stereoscopic condition is consistent with previous studies, indicating that a more accurate stereoscopic perception of locomotion might indeed be utilised in the flow parsing process.

A radial flow vector is ambiguous in a three-dimensional space. Such a flow vector at eye-level can infer both lateral and sagittal object motions. Binocular disparity provides additional motion-in-depth cues [26], potentially aids in the motion perception process.

Surprisingly, the sensitivity, or precision, was worse in the stereoscopic condition, inconsistent with previous research which suggested that precision is often better in binocular conditions [27–29].

## Experiment 3

One drawback to the stimulus of experiment 2 was that sometimes the target entered the blind spot of one eye, more likely during forward locomotion. This means in some trials the target might only be visible to one eye in the synoptic and the stereoscopic conditions, or could not be seen at all in the monocular condition, which was undesirable. In experiment 3, we fixed this by changing the initial target eccentricity to 8.5°. Under this eccentricity, the target was kept out of the blind spot (retinal motion was mostly < 3°).

Moreover, experiment 3 also offered a chance to reproduce the results of experiment 2 at a smaller target eccentricity. There is evidence that optic flow is processed differently at different eccentricities [4, 30–32]. When eccentricity increases, targets were harder to detect [32], the sensitivity to radial optic flow was found to decrease [30], and perceived inward target motion was found to be greater than outward motion [4]. Experiment 3 could help us examine whether the results of Experiment 2 still hold when the target eccentricity changes, including the opposite effects that binocular disparity had on bias and on precision.

### Materials and methods

#### Participants

Twelve participants, aged 19–55 (M = 31.58, SD = 9.86, seven males, five females) were recruited the same way as in Experiment 2, from 1 June 2022 to 31 August 2022. Among them, nine people participated in experiment 1 and seven participated in experiment 2.

#### Stimuli and procedure

Experiment 3 was identical to experiment 2 except that the eccentricity was at 8.5°.

### Results

**Fig 11:**
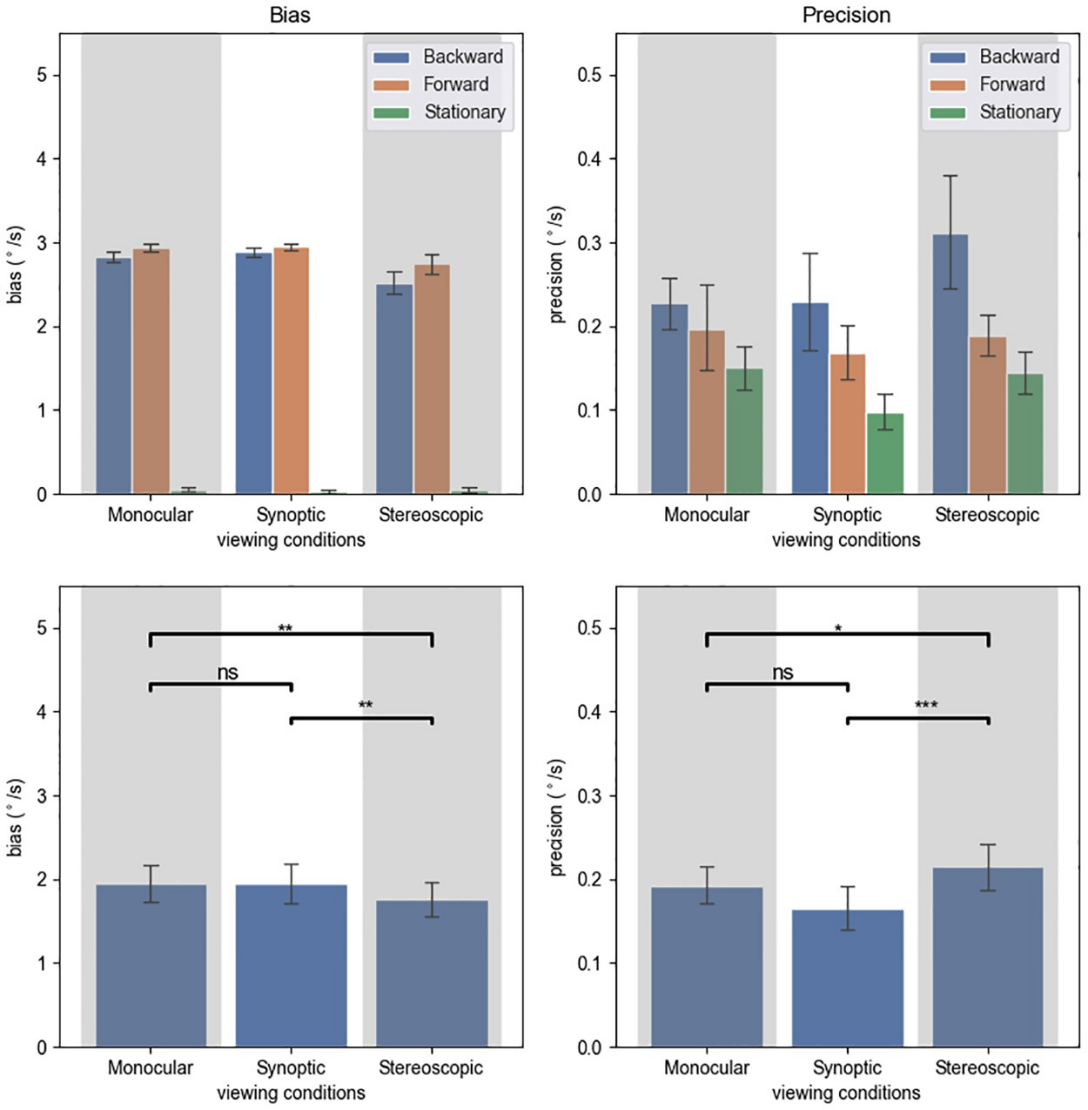
Experiment 3 results by viewing conditions. Top left: biases in angular unit (°/s), which were calculated by taking the average of the 29% and 71% thresholds. Top right: precision, obtained by taking half of the differences between the 29% and 71% thresholds. Bottom: biases and precisions by viewing conditions, with significance annotations. “*”: .01 ≤ p < .05, “**”: .001 ≤ p < .01, “***”: p < .001.

The results are shown in Fig 11. The data analysis steps were the same as in experiment 2.

#### Effects of locomotion direction

The effect of locomotion direction on bias can be observed in Fig 11. The repeated measures ANOVA revealed a significant main effect of locomotion direction (F(2,22) = 1152.381, p < .001, $\eta_{p}^{2}=0.991$). Specifically, the stationary bias was very close to zero, much lower than the backward bias(t(35) = 45.920, *p*_*adj*_ < .001), and the backward bias was slightly (4.52%) lower than the forward bias (t(35) = 2.755, *p*_*adj*_ < .009).

The ANOVA also showed a main effect of locomotion on precision (F(2,22) = 5.334, p = .013, $\eta_{p}^{2}=0.327$). The stationary precision was lower (better) than the forward precision (t(35) = 2.549, *p*_*adj*_ < .031), and the forward precision was lower (better) than the backward precision (t(35) = 2.310, *p*_*adj*_ < .031).

#### Effects of viewing condition

Similar to experiment 2, we performed t-tests to compare between the viewing conditions to examine our main hypotheses. The stereoscopic bias was lower than both the monocular bias (t(35) = 3.384, *p*_*adj*_ = .005) and the synoptic bias (t(35) = 3.300, *p*_*adj*_ = .005). The synoptic bias and the monocular bias were not significantly different (t(35) = .414, *p*_*adj*_ = .681).

The stereoscopic precision was not significantly different from the monocular precision (t(35) = 0.877, *p*_*adj*_ = .582), but significantly worse (24.12% higher) than the synoptic precision (t(35) = 4.055, *p*_*adj*_ < .001). The t-test did not find a significant difference between the monocular and the synoptic precision (t(35) = 1.073, *p*_*adj*_ = .582).

We also performed ANOVA. There was a significant effect of viewing condition on bias (F(2,22) = 7.483, p < .001, $\eta_{p}^{2}=0.405$). The ANOVA did not find a significant simple effect of viewing condition on precision (F(1.27,13.97) = 2.037, p = .175, $\eta_{p}^{2}=0.156$).

#### Interaction effects between locomotion and viewing conditions

A significant interaction effect on bias was found between locomotion and viewing condition (F(4,44) = 5.402, p = .001, $\eta_{p}^{2}=0.329$). We conducted subsidiary ANOVA for each locomotion direction. The effect of viewing condition was significant during backward locomotion (F(2,22) = 10.666, p < .001, $\eta_{p}^{2}=0.492$), and not significant during forward locomotion (F(1.16,12.79) = 3.267, p = .090, $\eta_{p}^{2}=0.229$) or when stationary (F(2,22) = .502, p = .612, $\eta_{p}^{2}=0.044$). Post-hoc t-tests showed that during backward locomotion, the monocular bias was significantly higher than the stereoscopic bias (t(11) = 3.028, *p*_*adj*_ = .023), not significantly different from the synoptic bias (t(11) = -0.969, *p*_*adj*_ = .353), and the stereoscopic bias was significantly lower than the stereoscopic bias (t(11) = -3.997, *p*_*adj*_ = .006).

There was no significant interaction effect between locomotion and viewing condition on precision (F(2.29,25.21) = 1.181, p = .328, $\eta_{p}^{2}=0.097$).

### Discussion

In Experiment 3, the stereoscopic bias was lower than the synoptic bias, and the synoptic bias was not significantly different from the monocular bias. This means that we found evidence that binocular disparity decreased the flow parsing bias, while binocular summation did not change the bias significantly. These effects were the same as experiment 2.

There are some minor differences between the results of experiment 2 and the results of experiment 3. An example is that the difference between the stereoscopic precision and synoptic precision in experiment 2 was not significant. In experiment 3, this difference was significant. This type of difference was not too surprising, given that they were different experiments and tested different participants.

Furthermore, bias was much smaller in experiment 3. This is consistent with previous finding that the bias increases as target eccentricity increases [33].

To conclude, experiment 3 suggested our main finding, that binocular disparity decreased flow parsing bias and worsened precision (while binocular summation did not change the bias or precision significantly), still holds true with a smaller target eccentricity. Results of experiment 3 also indicated that the blind spot was unlikely to have caused a major impact on the results of experiment 2.

## General discussion

### Binocular disparity and binocular summation

The discrimination biases in experiment 2 and 3 were both improved by binocular disparity. The detection threshold in experiment 1 was improved by binocular disparity only during locomotion.

In experiment 1, a useful strategy would be to compare the four targets and to look for the one that stands out (differs). Monocular cues are effective in this task, and since binocular summation strengthens monocular cues, the performance was improved by binocular summation when stationary. During locomotion, the distance becomes more relevant, as the observer needs to factor the self-motion component of the target motion to obtain scene-relative target motion (i.e. flow parsing). Binocular disparity is a cue to motion in depth [26]. This might explain why binocular disparity rather than binocular summation aids in this process.

In experiment 2 and experiment 3, binocular disparity significantly decreased the motion discrimination biases but binocular summation did not. Stereopsis might provide more accurate depth between the target and the background. The binocular disparity-defined distance could be utilised in the flow parsing process [3], which may explain the smaller stereoscopic bias. The binocular motion-in-depth signals [26] can describe the sagittal-parallel target movement more explicitly, such that when combined with monocular cues, there might be less flow vector component which was incorrectly mapped to a lateral target motion. Alternatively, it was possible that the stereoscopic condition provided a improved locomotion perception [14], lowering the bias.

An important motivation of our experiment was that the stimulus of Warren and Rushton [3] was carefully chosen so that the observers were forced to rely on binocular disparity, and we wanted to know if the visual system still relies on binocular disparity when other monocular cues are available. Our stimulus was different in (1) our target’s retinal size and direction changed in accordance with its distance and movement, (2) we used a more realistic scene, and (3) the target had a non-zero retinal flow vector. These signals are monocularly available and should be strengthened by binocular summation. However, we only observed an improved performance between the synoptic condition and the monocular condition in experiment 1, but not in experiments 2 or 3. This could mean that these monocular cues were more relied on in detection tasks than in discrimination tasks, and binocular disparity was more relied on in discrimination tasks than in detection tasks.

It is worth noting that even in the stereoscopic condition, the bias was relatively large compared to the locomotor speed during locomotion. This means flow-parsing tasks may be difficult in VR environments, even in a scene that looks more realistic than kinematograms.

### Binocular disparity did not improve motion discrimination precision

Previous research concluded that the precision of stereoscopic depth judgement is better than that of monocular depth judgements [27–29]. The precision results of experiment 1 were consistent with the previous research, while the results of experiment 2 and experiment 3 indicated otherwise. How could binocular disparity possibly hamper precision? The percept under monocular and synoptic conditions was biased. The decrease in precision could be the result of an unexpected addition of the correct binocular disparity.

We discussed in the discussion section for experiment 2 how binocular disparity could indicate a closer target distance by indicating a more evident depth difference between the target and background. Although binocular disparity can help in estimating distances, it does not provide absolute distance [15]. Inherently, the change in disparity (or the interocular velocity difference) does not provide a precise change in absolute distance. A visual system which uses binocular disparity to estimate distance suffers from this non-determinacy. However, in the synoptic condition, all disparity is zero, so disparity change is also zero. This might explain the slight sensitivity drop in the stereoscopic condition. Also, it might serve as evidence that the visual system tries to utilise binocular disparity to solve for flow parsing, and it prioritises correctiveness rather than precision.

The reason that experiment 1 was exempted from these confusions could be a difference in strategy. In experiment 1, there were four targets, where three were stationary and one was moving. Thus, both moving and stationary examples were given. Neither a specific speed estimation nor distance estimation of the targets was required, and the observers only needed to compare the relative movements among the examples. In experiments 2 and 3, there was only one target presented at a time. Thus, observers could not directly obtain a veridical reference of a stationary target from the stimulus, and they had to generate one themselves.

## Summary

We compared target motion detection and discrimination performance during locomotion in three viewing conditions to examine the role of binocular disparity and binocular summation in this process. Experiment 1 found interactions. When stationary, binocular summation significantly improved the detection threshold, but binocular disparity did not. During forward locomotion, binocular disparity significantly improved the detection threshold, but binocular summation did not. Experiment 2 and 3 showed that binocular disparity significantly decreased the discrimination bias, but binocular summation did not. Moreover, stereoscopic precision was worse than monocular and synoptic precisions, possibly because a non-zero depth between the target and the background was indicated by binocular disparity.

## Supporting information

S1 Video Sample video for experiment 1. Shows sample trials of Experiment 1. Note that the sample videos are modified and designed to be viewed from conventional devices such as a computer monitor or a smartphone, so they do not differentiate the stereoscopic, synoptic or monocular viewing conditions or mirror the experimental field of view.
(MKV)

S2 Video Sample video for experiment 2. Shows sample trials of Experiment 2.
(MKV)

S3 Video Sample video for experiment 3. Shows sample trials of Experiment 3.
(MKV)
